# Age patterns of suicide with different methods for US Whites: APC modelling analysis of the 1999–2017 national data

**DOI:** 10.1017/S204579602000092X

**Published:** 2020-11-13

**Authors:** B. Yu, X. Chen

**Affiliations:** 1 Department of Epidemiology, University of Florida, Gainesville, FL 32610, USA; 2 Department of Surgery, School of Medicine, Duke University, Durham, NC 27705, USA

**Keywords:** Age pattern, period–cohort adjusted rate, suicide, suicide methods

## Abstract

**Aims:**

Suicide emerges as a threat to national health of USA with Whites being at extra risk. More information is needed regarding the increased suicide among Whites to improve national suicide prevention strategies. This study aims to characterise the age pattern of suicide among Whites by suicide methods adjusting for time period and birth cohort.

**Methods:**

Suicide mortality data by age of 15–84 years during 1999–2017 were derived from the Wide-Ranging Online Data for Epidemiological Research, prepared by US Center for Disease Control and Prevention. Mortality data for three common suicide methods, firearms, suffocation and poisoning were analysed using the age–period–cohort (APC) model. Period–cohort adjusted mortality rates by age were estimated based on results from APC modelling.

**Results:**

Period–cohort adjusted rates indicated that the overall age pattern for males contained five phases, including three increasing phases (ages 15–20, 30–50 and 65+), connected by two declining phases (ages 20–30 and 50–65); and the age pattern for females was a parabolic with an increasing phase from 15 years of age up to 50, followed by a declining phase after age 50. Furthermore, the age pattern for different suicide methods differed substantially for males, but did not for females. Among males, suicide by firearms contained two rapid increasing phases, one during adolescence and another in older ages; suicide by suffocation showed a high plateau across an age span from 20 to 55 years; and suicide by poisoning followed a parabolic, increasing by age up to 45 before it declined. Age patterns revealed by the unadjusted crude rates were biased because of significant linear period effect and W-shaped cohort effect.

**Conclusions:**

This study is the first to quantify the age patterns of suicide by different methods for US Whites using period–cohort adjusted rates. Study findings provide valid evidence supporting precision interventions to reduce the extra suicide mortality among Whites by targeting specific age ranges with different suicide methods.

## Introduction

National strategies to curb the growing trend of suicide among US population require unbiased measure of the risk and pattern of suicide by different methods for planning and decision-making. Data from the Centers for Disease Control and Prevention (CDC) indicate that suicide is the 10th leading cause of death in the USA with a total of 47 173 persons killed themselves in 2017 alone (AFSP, 2017). According to the National Vital Statistics System, the age-standardised suicide mortality per 100 000 increased 33% from 10.5 in 1999 to 14.0 in 2017 (CDC, 2018). Relative to non-Whites, Whites are more likely to die by suicide, accounting for more than 70% of the national total (AFSP, 2017).

Firearms are the most commonly used method for suicide in the USA, accounting for 50.57% of the total suicide deaths; followed by suffocation (including hangings), and poisoning (AFSP, 2017). In addition to the overall differences, valid data on age patterns of different suicide methods are crucial. Such data will provide information much needed for evidence-based planning and preparation of targeted prevention interventions and relevant clinical services for emergency treatment to reduce suicide (Doshi *et al*., 2005; Snowdon *et al*., 2017; CDC, 2019). However, unbiased data are lacking on the risk of suicide by age for different suicide methods. A primary goal of the current study is to fill this gap.

It is a challenge to determine the age pattern of suicide risk epidemiologically because the directly computed suicide rates by age could be biased due to changes in the risk over time periods and variations in risk across birth cohorts (Yang and Land, 2013; Chen *et al*., 2018; Yu and Chen, 2019). With multi-year national data, findings from previous studies indicate significant impact of time period and birth cohort on the risk of suicide by age (Phillips, 2014; Wang *et al*., 2016; Chen *et al*., 2018; Yu and Chen, 2019). For example, one study indicated a greater risk of suicide by ages during 1975–1979 than the other years (Phillips, 2014); and another study reported that individuals born after 1995 experienced increased risk of suicide (Yu and Chen, 2019). Observed age patterns of the risk of suicide will be biased without considering the impacts of time period and birth cohort. Fortunately, unbiased rates can be obtained using the classic age–period–cohort (APC) model (Yang and Land, 2013; O'Brien, 2014), as we demonstrated in another suicide study (Yu and Chen, 2019). Here, this study applies the APC modelling framework to obtain unbiased estimates to determine the risk of suicide by age, overall and stratified by different suicide methods.

## Materials and methods

### Data source

De-identified data for Whites were derived from the Wide-Ranging Online Data for Epidemiologic Research (WONDER), a data source sponsored by CDC for data sharing (CDC, 2020). Suicide events were defined using the International Statistical Classification of Diseases and Related Health Problems, 10th edition (ICD-10), and deaths coded as U03, X60–X84 and Y87.0 during 1999–2017 were included. Data used for this study included total population size, number of suicide deaths, suicide methods (e.g. firearm, suffocation and poisoning), age (in years) at suicide death and sex (male/female). Annual data from 1999 to 2017 were used. Suicide deaths outside of the age range of 15–84 years were few. These subjects were excluded to ensure robustness of parameter estimates in APC modelling analysis.

Approval of this study was obtained from the Institutional Review Board at specific institute.

### Measurement

Three independent variables used in the APC modelling included: chronological age (year), time period (calendar year of suicide death) and birth cohort (calendar year of birth). (a) The variable chronological age was measured as the registered age when suicide death was reported (range: 15–84 years). (b) The variable time period was measured using the calendar year when a suicide death was reported (range: 1999–2017). (c) The variable birth cohort was computed by subtracting chronological age from time period (range: 1915–2002).

### APC modelling analysis

First, annual crude suicide rates (per 100 000) by age from 1999 to 2017 were computed as the number of suicide deaths divided by the population estimates. The computed crude rates were then used as the outcome variable to link with the three independent variables age, period and cohort using the following APC model:1
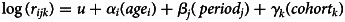
where *r*_*ijk*_ represents the crude suicide rates for individual subjects in age *i*, period *j* and cohort *k*; *u* is the intercept or grand mean; *α*_*i*_ denote the effect from chronological age *i* (*i* = 15, 16, 17, …, 84); *β*_*j*_ is the effect for time period *j* (*j* = 1999, 2000, 2001, …, 2017) and *γ*_*k*_ is the effect for birth cohort *k* (*k* = 1915, 1916, …, 2002).

To address the collinearity between the age, period and cohort effect (i.e. Cohort = Period − Age), the intrinsic estimator (IE) method (Yang and Land, 2013) was used to estimate parameters of the defined APC model in Equation (1). IE was developed based on the singular value decomposition and estimable functions to uniquely determine the estimates of APC model parameters. The computational algorithm of IE is an orthonormal transformation of the principal components regression (Yang *et al*., 2008; Yang and Land, 2013).

In APC modelling analyses, suicide death as a rare event was assumed to follow a Poison distribution. The modelling analysis was implemented in statistical software Stata (version 15.0) by calling the package ‘apc’ that is specifically developed for parameter estimation using the IE method (Yang and Land, 2013).

### Estimation of adjusted rate by age

Since the estimated age, period and cohort effect *α*_*i*_, *β*_*j*_ and *γ*_*k*_ are statistically independent from each other, the estimated age effect *α*_*i*_ provides an unbiased measure of the risk of suicide by age after controlling for the impact of period and cohort. With the estimated age effect *α*_*i*_ from data, period–cohort adjusted suicide mortality rate was estimated using the following equation:2
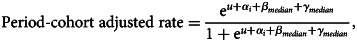
where *u* and *α*_*i*_ (*i* = 15, 16, …, 84) are the same as in Equation (1); *β*_*median*_ and *γ*_*median*_ are the median of the estimated period and cohort effects, all being estimated using Equation (1). After controlling for period and cohort, the adjusted rates by age provide an unbiased measure of age patterns of suicide. Therefore, period–cohort adjusted rates are more valid than unadjusted crude rates to quantify the age pattern of suicide.

In presenting the results, estimated age effects and period–cohort adjusted rates were plotted by age to visually reveal the age patterns of suicide risk. The period–cohort adjusted rates and unadjusted rates were plotted together to visualise the impact (confounding effect) on age pattern after removal of the period and cohort effects. Plots were made for individual suicide methods, overall and stratified by sex of male and female. In presenting the study findings by age, 2-year average was used to remove minor fluctuations in the estimates.

## Results

### Crude rates of age-specific suicide mortality

Table 1 presents crude rates of suicide mortality by 5-year age interval for the three suicide methods by sex during 1999–2017. The crude rates among males increased from age 15–19 to 50–54 years, followed by a decline to 65–69 years, and increased again until 80–84 years. Firearm was the most common method of suicide among males followed by suffocation and poisoning.

**Table 1.** Age-specific suicide mortality among US Whites, overall, by sex and by methods, 15–84 years old, 1999–2017

Age	Male				Female			
	Total	Firearm	Suffocation	Poisoning	Total	Firearm	Suffocation	Poisoning
15–19	15.19	8.10	5.36	0.78	3.82	1.10	1.80	0.59
20–24	26.12	14.19	8.22	1.87	4.94	1.67	1.71	1.11
25–29	27.47	14.10	8.85	2.65	6.27	2.16	1.84	1.77
30–34	27.95	13.72	8.89	3.45	7.51	2.57	1.92	2.45
35–39	29.58	14.51	8.64	4.34	8.86	2.89	1.85	3.43
40–44	31.61	15.84	8.25	5.24	10.41	3.40	1.82	4.37
45–49	33.41	17.47	7.71	5.70	11.28	3.68	1.74	4.96
50–54	33.74	19.03	6.78	5.47	11.34	3.75	1.61	5.04
55–59	31.98	19.56	5.42	4.78	10.12	3.42	1.33	4.45
60–64	27.89	18.91	3.79	3.34	7.95	2.90	0.97	3.37
65–69	26.21	19.85	2.58	2.35	6.10	2.39	0.73	2.41
70–74	29.68	24.05	2.21	2.11	4.97	2.08	0.54	1.85
75–79	35.80	30.01	2.35	2.03	4.61	1.85	0.64	1.66
80–84	43.79	36.43	3.01	2.49	4.31	1.53	0.72	1.52
Total	29.20	17.22	6.47	3.59	7.81	2.66	1.47	3.01

*Note*: Data were derived from CDC WONDER (https://wonder.cdc.gov/).

Among females, the crude rates also increased from age 15–19 to 50–54 years, but followed by declining trend all the way to the last age group. Poisoning was the top one methods of suicide, followed by firearm and suffocation.

### Age, period and cohort effects from APC modelling

**Fig. 1.**
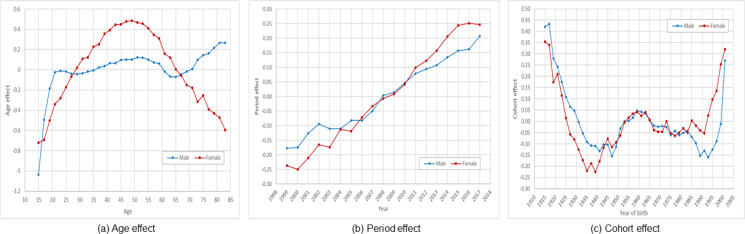
Estimated age, period and cohort effects for US Whites, 15–84 years, 1999–2017. *Data source*: CDC WONDER (https://wonder.cdc.gov/).

Figure 1a indicates substantial sex differences in the pattern of suicide risk by age. The pattern for males contained five phases, including three increasing phases (ages 15–20, 30–50 and 65+), connected by two declining phases (ages 20–30 and 50–65). The pattern for females differed from that for males. The risk for females showed a parabolic pattern with a gradual increase from age 15 to 50, followed by a progressive decline thereafter.

The estimated period and cohort effects are presented in Figs 1b and 1c. As shown in the figures, there were substantial linear increasing period effect and W-shaped cohort effect. The observed period and cohort effect support our approach to describe the risk of suicide by age using period–cohort adjusted rates.

### Age pattern of suicide by suicide methods among White males

**Fig. 2.**
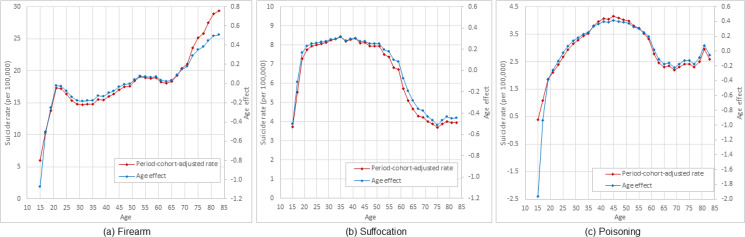
Period–cohort adjusted age-specific rate and APC model estimated age effect of suicide by methods among US White males, 15–84 years old, 1999–2017. *Note*: Estimated using 1999–2017 national mortality data from CDC WONDER (https://wonder.cdc.gov/).

The estimated age effect and adjusted rates in Fig. 2a indicate that the age pattern of suicide deaths using firearms was similar to the pattern of total population as shown in Fig. 1a – a three-phase pattern with two quick increasing phases in age ranges of 15–20 years and 65–84 years, and a fluctuation in age range of 20–65 years.

Similarly, Fig. 2b presents estimated age effect and adjusted suicide rates using the suffocation method. Data in the figure show a totally different age pattern of suicide by suffocation from that by firearms in Fig. 2a. There were still three phases, including a similar rapid increasing phase during 15–20 years and a plateau phase between 20 and 55 years; but ended with a 30-year long declining phase up to 75 years of age and a small increase thereafter.

Figure 2c presents the age pattern of suicide by poisoning using the estimated age effects as well as the adjusted suicide mortality rates. The results indicate a rapid increasing phase from 15 years of age to peak at age 45 years, followed by a declining phase from 45 to 66 years old, and ended a small increase plus some fluctuations.

### Age pattern of suicide by suicide methods among White females

**Fig. 3.**
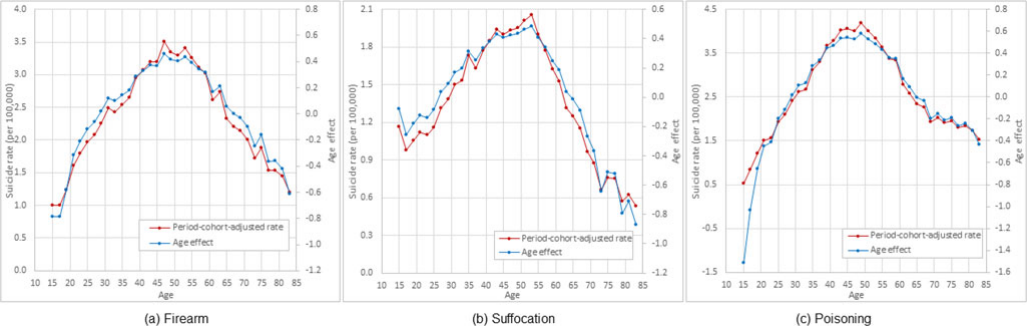
Period–cohort adjusted age-specific rate and APC model estimated age effect of suicide by methods among US White females, 15–84 years old, 1999–2017. *Note*: Estimated using 1999–2017 national mortality data from CDC WONDER (https://wonder.cdc.gov/).

Figure 3 presents the estimated age effect and adjusted suicide mortality rates by age for suicide by firearms (Fig. 3a), suffocation (Fig. 3b) and poisoning (Fig. 3c). Results in the figure indicate a similar two-phase pattern for all three suicide methods: a rapid increasing phase in the age range 15–50 years, followed by a declining phase thereafter.

### Comparison of the period–cohort adjusted and unadjusted rates

**Fig. 4.**
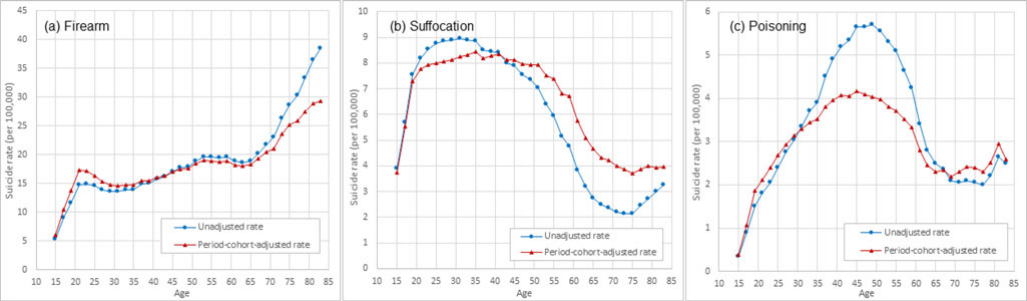
Unadjusted and period–cohort adjusted age-specific rate of suicide by methods among US White males, 15–84 years old, 1999–2017. *Note*: Estimated using 1999–2017 national mortality data from CDC WONDER (https://wonder.cdc.gov/).

**Fig. 5.**
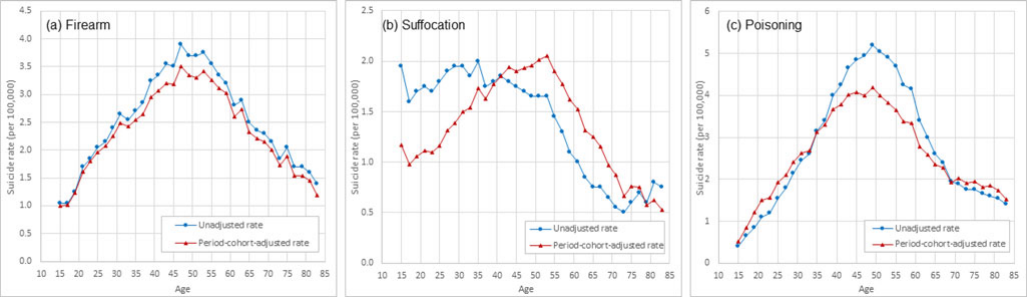
Unadjusted and period–cohort adjusted age-specific rate of suicide by methods among US White females, 15–84 years old, 1999–2017. *Note*: Estimated using 1999–2017 national mortality data from CDC WONDER (https://wonder.cdc.gov/).

Figures 4 and 5 present the estimated period–cohort adjusted rate and unadjusted rate of suicide by sex and by suicide methods. Results in Fig. 4 indicate that among males, the adjusted suicide rate by firearm was higher than the unadjusted rate during ages 15–45 years then lower after 45 years; the adjusted rate by suffocation was lower than the unadjusted rate during ages 20–40 years, and higher after 40 years; the adjusted rate by poisoning was higher than the unadjusted rate in ages 15–30 and 67–83 years, and lower during 30–67 years old.

Results in Fig. 5 indicate that among females, the adjusted rate of suicide by firearm was overall lower than unadjusted during 15–84 years; the adjusted suicide rate by suffocation was lower than the unadjusted rate in the ages 15–41 and after 79 years, and higher during 41–79 years of age; the adjusted suicide rate by poisoning was higher than the unadjusted among those aged 15–35 and 69–83 years, and lower during 35–69 years of age.

## Discussion

This study examined the age pattern of suicide mortality risk for US Whites aged 15–84 years by sex, and by suicide methods of firearms, suffocation and poisoning. The study used national data during 1999–2017, documented by the National Vital Statistical System. The period–cohort adjusted suicide rates by age were obtained using the APC modelling capable of controlling for potential bias due to changes in risk over time periods when suicide occurred and in birth year of individual subjects. The adjusted and unbiased rates by age provide information better than the crude rates to inform evidence-based decision-making and precision prevention intervention programmes, and relevant clinical treatment to reduce the extra risk of suicide deaths among Whites, and suicide death in the USA in general.

### Age patterns of suicide among US Whites

Based on the age effect derived from APC modelling, the overall age pattern for White males contains five phases, including three increasing phases (ages 15–20, 30–50 and 65+), connected by two declining phases (ages 20–30 and 50–65). This age pattern corresponds to the general life course milestones. Although investigation of the underlying causes of the observed age pattern is beyond the scope of the study, possible explanations may include the following:

For the three phases of increased risk, (1) the increased suicide risk in the early 20s may be associated with the imbalance of cognitive and physical development of puberty. During this period, young males leave home and go to colleges and universities. They are thus physically becoming more independent from parents, but cognitively may not be ready for independent life (Chen *et al*., 2017; Yu and Chen, 2019). (2) The small increase in suicide risk from 30 to 50 years of age may be related to greater strains in daily life, including marriage, family, employment and career (Bohnert *et al*., 2011) and more frequent mental health problems (Kessler *et al*., 2010). (3) For males aged 65 years and older, many factors may contributed to the increased risk of suicide, including retirement-related social disconnection and isolation, economic insecurity, losing partners, and physical and mental health illness, and greater access to suicide methods (Conwell *et al*., 2002; Conejero *et al*., 2018).

For the two phases with declining suicide risk, the age ranges correspond to the time periods when young adults aged 20–30 step into society with hope and enthusiasm and when middle-aged adults in 50–65 with lifetime achievement and prepare for their retirement life. These life events are associated with less stress (Girard, 1993), therefore reducing the risk of suicide.

The age pattern for White females is parabolic with an increasing phase from 15 years of age up to 50 years, followed by a declining phase thereafter. These results suggest that despite increases in formal employment, families and children are the main focus for females. Up to age 50, the major life events for females in the USA include receiving education, getting married, taking care of children and experiencing empty nest syndrome when children grow up, and adjustment for menopause (Girard, 1993; Grzywacz *et al*., 2002; Phillips, 2014). There are increased stresses and strains associated with these life events for females (Girard, 1993).

After 50 years, most females may shift their attention from family and children to themselves, leading to progressive declines in strains and stress, manifested as declines in the risk of suicide (Girard, 1993). Other supportive evidence is lower prevalence of depression for females 50 years of age and older relative to the younger females (Kessler *et al*., 2010). Furthermore, different from males, females who have not been employed will not experience any retirement-related stress; while females who have been employed, they are more likely than males to maintain social ties and to build new social ties after retirement, increasing their resilience against suicide (Conejero *et al*., 2018).

Findings of the study also indicate that compared to males, females have a much lower risk of suicide across all ages. This is consistent with findings from previous studies in the USA (Phillips, 2014; Yu and Chen, 2019) and many other countries across the world (Bachmann, 2018). This sex difference may be attributed to the fact that males are more likely to commit suicide with highly lethal methods than females (Kumar and Mandal, 2010), although suicidal behaviours are more prevalence among females (AFSP, 2017).

### Prevention of suicide among White males

First, the risk of suicide by firearms among males showed a quick increase in two age ranges, 15–20 and 65–84 years old, connected by a sigmoid-like progression from age 20 to 65 years. This age pattern provides evidence for policy and decision-makers, researchers and clinicians to devise and implement prevention programmes and treatment services better targeting the firearm-related suicide. The two critical periods for preventing and treating this type of suicide are before age 20 and after age 65. Examples of possible interventions include limiting firearm purchases, such as extending the waiting period for purchasing firearms for youth and elderly, implementing the Child Access Prevention Programmes for youth and Extreme Risk Protection Orders Programmes for elderly and creating stricter regulations for storage of firearms in homes (Cantor and Slater, 1995; Miller and Hemenway, 2001; Rodríguez Andrés and Hempstead, 2011; Giffords Law Center to Prevent Gun Violence, 2019a; 2019b). Epidemiologists, pediatricians, psychiatrists and other clinical and public health workers must also enhance the surveillance of the rapid increases in suicide in young Whites up to age 20.

Second, suicide by suffocation among White males showed a four-stage age pattern: a rapid increase stage from 15 to 20 years of age, a stable stage from 20 to 55, a dramatic decline stage from 55 to 75 and ended with a small increase stage from 75 to 84. This pattern was distinctly different from that of suicide by firearms. This pattern shows that great attention should be paid to young males with a rapid increase in suffocation with age and the high-risk age range up to 55 years old. Suffocation, particularly hanging, is highly accessible and very easy to implement. It is very challenging to restrict the access to means of suffocation with limited effect (Gunnell *et al*., 2005; Baker *et al*., 2013). Our findings support the significance of earlier interventions to treat mental health problems among young White males (Kosky and Dundas, 2000). More research studies are needed to prevent suicide by suffocation, particularly among young White males.

Third, suicide by poisoning for White males showed a three-stage pattern that was different from the other two methods. It includes: a rapid increase stage (15–45), a quick declining stage (45–70) and a stable stage with a small increase (70–84). The risk of suicide by poisoning increased the quickest and lasted the longest age range relative to the other two methods of suicide. The risk does not decline until 45 years of age. Of the suicide deaths by poisoning, a majority are due to substance overdose, especially overdose of opioids (CDC Injury Center, 2019). Consistent with our findings, previous studies indicate that over 80% of opioid overdose deaths happened in persons aged 40–60 years (Bohnert *et al*., 2011). Meanwhile, people in this age range have easier access to opioids, and may use opioids to cope with the chronic illness, life strains and mental health problems, increasing their risk of substance overdose death (Bohnert *et al*., 2011). Our finding provide concrete evidence at the population level supporting the existing prevention strategies on opioid use starting at young adulthood, including doctors’ responsible opioid prescribing, reducing individuals’ exposure to opioids, preventing opioids misuse and treating opioids use disorders (CDC Injury Center, 2017).

### Prevention of suicide among White females

Compared to males, age patterns of suicide risk for females are less complex. Risk of suicide by age for all the three suicide methods follows a parabolic pattern with a dramatic increase until age 50, followed by a decline until age 84. The main differences include a somewhat symmetric for firearms; a higher starting level and quick decline for suffocation; and a very lower starting level with a rapid increase to 50 years of age, followed by a gradual decline for poisoning.

The adjusted rates indicate that suicide risk for White females from high to low are poisoning, firearm and suffocation. This result is consistent with study findings by others that females prefer less lethal methods than males (Callanan and Davis, 2012). Based on the parabolic age pattern, strategy of suicide prevention among White females in the USA should start in the early 20s and continue up to 40–50 years of age when most of them pass the high-risk age range. The parabolic pattern of suicide risk by age also informs decision-makers, psychiatrists and community health workers to improve their work to reduce White suicide disparities among US females. For example, it would be effective for mental health service to specifically target prescribing drugs abuse, especially opioids abuse among females (Thornicroft and Tansella, 2004; Wheeler *et al*., 2012; Compton *et al*., 2015). To further understand the age pattern, more individual-level data are needed to investigate the factors and underlying mechanisms associated with the increasing trend before 50 years and the decline thereafter.

### Period and cohort effects of suicide mortality

Findings of the study reveal substantial differences between the period–cohort adjusted and unadjusted rates of suicide by age. Furthermore, these differences are attributed to the time period and birth cohort (Yu and Chen, 2019). For suicide mortality among US Whites, there is a linear increase in the suicide risk during the study period during 1999–2017 and a W-shaped cohort effect during a historical period of 88 years from 1915 to 2002. Consistent with findings from previous studies (Phillips, 2014; Wang *et al*., 2016; Yu and Chen, 2019), there is an increased risk of suicide in the USA since 1999 with no signs of decline. Further studies are needed to investigate the increasing trend in suicide risk among US Whites.

The W-shaped cohort effect provides new information regarding suicide risk in the USA since 1915. This finding underscores the need for further investigation of this risk pattern from a historical epidemiological perspective to shed light on current trend of suicide as reported in other studies on population mortality in general (Chen and Wang, 2014) and suicide mortality in particular (Chen *et al*., 2018; Yu and Chen, 2019). For example, such studies may provide evidence supporting the suicide prevention for US Whites who were born after 1995, corresponding to the Internet-Generation. Most males and females of this generation are characterised with long-term exposure to internet and social media that may increase risk of suicide (Messias *et al*., 2011; Luxton *et al*., 2012; Twenge, 2017; Yu and Chen, 2019).

## Limitations

This study has limitations. The purpose of this ecological study is to detect the mega age pattern of suicide death by different methods; and no research has conducted to analyse mechanisms and influential factors underlying the detected age patterns. This study is based on national data which only provide data for three suicide methods. This prevents us from investigating other suicide methods. Individual-level data with more detailed information are needed to address these issues in future studies. Despite the limitations, findings of the study add new knowledge to the suicide literature on extra burdens of suicide among US Whites. These findings also provide valid data supporting evidence-based programming for precision prevention and treatment to reduce suicide in the USA in general and White suicide disparities in particular.

## Data Availability

All data used in the study are publicly available through CDC WONDER website (https://wonder.cdc.gov/).

## References

[ref1] AFSP (2017) Suicide Statistics. https://afsp.org/about-suicide/suicide-statistics/ Accessed 13 March 2019.

[ref2] Bachmann S (2018) Epidemiology of suicide and the psychiatric perspective. International Journal of Environmental Research and Public Health 15, 1425. doi: 10.3390/ijerph15071425. PMC6068947 29986446

[ref3] Baker SP, Hu G, Wilcox HC and Baker TD (2013) Increase in suicide by hanging/suffocation in the U.S., 2000–2010. American Journal of Preventive Medicine 44, 146–149. 23332330 10.1016/j.amepre.2012.10.010 PMC3553495

[ref4] Bohnert ASB, Valenstein M, Bair MJ, Ganoczy D, McCarthy JF, Ilgen MA and Blow FC (2011) Association between opioid prescribing patterns and opioid overdose-related deaths. JAMA 305, 1315–1321. 21467284 10.1001/jama.2011.370

[ref5] Callanan VJ and Davis MS (2012) Gender differences in suicide methods. Social Psychiatry and Psychiatric Epidemiology 47, 857–869. doi: 10.1007/s00127-011-0393-5. 21604180

[ref6] Cantor CH and Slater PJ (1995) The impact of firearm control legislation on suicide in Queensland: preliminary findings. The Medical Journal of Australia 162, 583–585. doi: 10.5694/j.1326-5377.1995.tb138547.x. 7791644

[ref7] CDC (2018) Suicide Mortality in the United States, 1999–2017. https://www.cdc.gov/nchs/products/databriefs/db330.htm Accessed November 19 2019.

[ref8] CDC (2019) Prevention Strategies. https://www.cdc.gov/violenceprevention/suicide/prevention.html Accessed December 3 2019.

[ref9] CDC (2020) CDC WONDER. https://wonder.cdc.gov/ Accessed March 7 2020.

[ref10] CDC Injury Center (2017) Overdose Prevention. https://www.cdc.gov/drugoverdose/prevention/index.html Accessed November 27 2019.

[ref11] CDC Injury Center (2019) Drug overdose deaths. https://www.cdc.gov/drugoverdose/data/statedeaths.html Accessed October 8 2019.

[ref12] Chen X and Wang P (2014) Social changes in China and the dynamic change of national health. Chinese Journal of Population Science 2, 63–73.

[ref13] Chen X, Yu B, Lasopa S and Cottler LB (2017) Current patterns of marijuana use initiation by age among US adolescents and emerging adults: implications for intervention. American Journal of Drug and Alcohol Abuse 43, 261–270. 27211100 10.3109/00952990.2016.1165239

[ref14] Chen X, Sun Y, Li Z, Yu B, Gao G and Wang P (2018) Historical trends in suicide risk for the residents of mainland China: APC modeling of the archived national suicide mortality rates during 1987–2012. Social Psychiatry and Psychiatric Epidemiology 54, 99–110. 30171272 10.1007/s00127-018-1593-z

[ref15] Compton WM, Boyle M and Wargo E (2015) Prescription opioid abuse: problems and responses. Preventive Medicine 80, 5–9. 25871819 10.1016/j.ypmed.2015.04.003

[ref16] Conejero I, Olié E, Courtet P and Calati R (2018) Suicide in older adults: current perspectives. Clinical Interventions in Aging 13, 691–699. 29719381 10.2147/CIA.S130670 PMC5916258

[ref17] Conwell Y, Duberstein PR, Connor K, Eberly S, Cox C and Caine ED (2002) Access to firearms and risk for suicide in middle-aged and older adults. The American Journal of Geriatric Psychiatry 10, 407–416. 12095900

[ref18] Doshi A, Boudreaux ED, Wang N, Pelletier AJ and Camargo CA (2005) National study of US emergency department visits for attempted suicide and self-inflicted injury, 1997–2001. Annals of Emergence Medicine 46, 369–375. 10.1016/j.annemergmed.2005.04.018 16183394

[ref19] Giffords Law Center to Prevent Gun Violence (2019a) Waiting Periods. https://lawcenter.giffords.org/gun-laws/policy-areas/gun-sales/waiting-periods/ Accessed January 17, 2020.

[ref20] Giffords Law Center to Prevent Gun Violence (2019b) Child Access Prevention. https://lawcenter.giffords.org/gun-laws/policy-areas/child-consumer-safety/child-access-prevention/ Accessed January 17, 2020.

[ref21] Girard C (1993) Age, gender, and suicide: a cross-national analysis. American Sociological Review 58, 553.

[ref22] Grzywacz JG, Almeida DM and McDonald DA (2002) Work-family spillover and daily reports of work and family stress in the adult labor force. Family Relations 51, 28–36.

[ref23] Gunnell D, Bennewith O, Hawton K, Simkin S and Kapur N (2005) The epidemiology and prevention of suicide by hanging: a systematic review. International Journal of Epidemiology 34, 433–442. 15659471 10.1093/ije/dyh398

[ref24] Kessler RC, Birnbaum H, Bromet E, Hwang I, Sampson N and Shahly V (2010) Age differences in major depression: results from the National Comorbidity Survey Replication (NCS-R). Psychological Medicine 40, 225–237. 19531277 10.1017/S0033291709990213 PMC2813515

[ref25] Kosky RJ and Dundas P (2000) Death by hanging: implications for prevention of an important method of youth suicide. Australian and New Zealand Journal of Psychiatry 34, 836–841. 11037371 10.1080/j.1440-1614.2000.00807.x

[ref26] Kumar U and Mandal MK (Eds.) (2010) Suicidal Behaviour: Assessment of People-At-Risk. India: Sage Publications.

[ref27] Luxton DD, June JD and Fairall JM (2012) Social media and suicide: a public health perspective. American Journal of Public Health 102(Suppl 2), S195–200. 22401525 10.2105/AJPH.2011.300608 PMC3477910

[ref28] Messias E, Castro J, Saini A, Usman M and Peeples D (2011) Sadness, suicide, and their association with video game and internet overuse among teens: results from the youth risk behavior survey 2007 and 2009. Suicide and Life-Threatening Behavior 41, 307–315. 21463355 10.1111/j.1943-278X.2011.00030.x

[ref29] Miller M and Hemenway D (2001) Firearm prevalence and the risk of suicide: a review. Harvard Health Policy Review 2, 29–37.

[ref30] O'Brien R (2014) Age-Period-Cohort Models: Approaches and Analyses with Aggregate Data. Boca Raton, Florida, USA: Chapman and Hall/CRC. doi:10.1201/b17286.

[ref31] Phillips JA (2014) A changing epidemiology of suicide? The influence of birth cohorts on suicide rates in the United States. Social Science & Medicine 114, 151–160. 24929916 10.1016/j.socscimed.2014.05.038

[ref32] Rodríguez Andrés A and Hempstead K (2011) Gun control and suicide: the impact of state firearm regulations in the United States, 1995–2004. Health Policy 101, 95–103. 21044804 10.1016/j.healthpol.2010.10.005

[ref33] Snowdon J, Phillips J, Zhong B, Yamauchi T, Chiu HFK and Conwell Y (2017) Changes in age patterns of suicide in Australia, the United States, Japan and Hong Kong. Journal of Affective Disorder 211, 12–19. 10.1016/j.jad.2017.01.007 28081432

[ref34] Thornicroft G and Tansella M (2004) Components of a modern mental health service: a pragmatic balance of community and hospital care: overview of systematic evidence. British Journal of Psychiatry 185, 283–290. 10.1192/bjp.185.4.283 15458987

[ref35] Twenge JM (2017) iGen: Why Today's Super-Connected Kids Are Growing Up Less Rebellious, More Tolerant, Less Happy – and Completely Unprepared for Adulthood – and What That Means for the Rest of Us. Simon and Schuster, New York, NY.

[ref36] Wang Z, Yu C, Wang J, Bao J, Gao X and Xiang H (2016) Age-period-cohort analysis of suicide mortality by gender among white and black Americans, 1983–2012. International Journal of Equity in Health 15, 107. 10.1186/s12939-016-0400-2 PMC4944259 27412030

[ref37] Wheeler E, Davidson PJ, Jones TS and Irwin KS (2012) Community-based opioid overdose prevention programs providing naloxone – United States, 2010. MMWR. Morbidity and Mortality Weekly Report 61, 101–105. 22337174 PMC4378715

[ref38] Yang Y and Land K (2013) Age-Period-Cohort Analysis: New Models, Methods, and Empirical Applications. Boca Raton, Florida, USA: Chapman and Hall/CRC. doi:10.1201/b13902.

[ref39] Yang Y, Schulhofer-Wohl S, Fu W and Land K (2008) The intrinsic estimator for age-period-cohort analysis: what it is and how to use it. American Journal of Sociology 113, 1697–1736.

[ref40] Yu B and Chen X (2019) Age and birth cohort-adjusted rates of suicide mortality among US male and female youths aged 10 to 19 years from 1999 to 2017. JAMA Network Open 2, e1911383. 31517968 10.1001/jamanetworkopen.2019.11383 PMC6745055

